# A cross-sectional study on diabetes epidemiology among people aged 40 years and above in Shenyang, China

**DOI:** 10.1038/s41598-020-74889-x

**Published:** 2020-10-20

**Authors:** Cong Liu, Xiaojiu Li, Muhui Lin, Limin Zheng, Xiaohong Chen

**Affiliations:** 1 Department of Neurology, The People’s Hospital of Liaoning Province, China Medical University, Shenyang, 110000 Liaoning China; 2 Department of Neurology, The People’s Hospital of Liaoning Province, Shenyang, 110000 Liaoning China; 3 Department of Neurology, Jin Qiu Hospital of Liaoning Province, Geriatric Hospital of Liaoning Province, Shenyang, 110000 Liaoning China

**Keywords:** Epidemiology, Population screening

## Abstract

This study aimed at understanding the diabetic prevalence, awareness, treatment and control rates and their influencing factors among people aged ≥ 40 years in Shenyang, China. A face-to-face cross-sectional epidemiological survey was conducted on the respondents using the national unified questionnaire. A total of 3922 respondents were enrolled, including 609 cases of diabetes. The diabetic prevalence rate was 15.5%, and was higher in rural areas than that in urban areas (17.7% vs*.* 14.2%, *p* = 0.004), while no difference was observed between men and women (14.8% vs*.* 16.1%, *p* = 0.242). Advanced age, hypertension and dyslipidemia were the diabetes influencing factors. Among the 609 respondents with diabetes, the diabetic awareness and treatment rates, and the control rate of fasting plasma glucose were 82.3%, 36.6% and 17.1%, respectively. In different age groups, the diabetic awareness rate was higher in men than that in women, and the treatment rate was higher in women than that in men. The diabetic patients, who consumed fruit for ≥ 5 days a week, accounted for 16.3%, and their diabetic treatment (28.1%) and control rates (44.1%) were lower. Shenyang people aged ≥ 40 years have higher diabetic prevalence and awareness rates, and lower diabetic treatment and control rates. Finally, it is necessary to enhance awareness and education about diabetes, to improve its treatment and control rates.

## Introduction

Cerebrovascular disease has become a major human health threatening disease^1^, and diabetes is an important risk factor. It is estimated that the number of diabetic patients will reach 552 million by 2030^2,3^. Diabetic eye disease and nephropathies are common diabetic complications, that can lead to blindness and renal failure^4,5^. In addition, diabetes is also significantly related to the occurrence, death and all-cause mortality of cerebrovascular diseases, making it a serious economic burden for individuals and society. In China, the diabetic prevalence rate is rising^6^, which may be due to diabetes lower awareness, treatment and control rates, and despite the improvement in people's living standards. Due to the economic, cultural, dietetic, and lifestyle influences, the prevention, diagnosis, and treatment of diabetes vary from region to region in China.

Shenyang is a high-prevalence area of cerebrovascular disease in the northeast of China, and there are currently no epidemiological surveys on adult diabetes in Shenyang. In this study, people aged ≥ 40 years, in Shenyang City communities and townships, Liaoning Province, were recruited as respondents, and diabetes prevalence, awareness, treatment, and blood glucose control rates (based on fasting plasma glucose, FPG) were investigated to provide a theoretical basis for diabetes prevention and treatment, and for the future development of stroke prevention strategies in Shenyang, Liaoning.

## Results

A total of 3949 people agreed to participate in the study, including 22 with incomplete data, and 5 without FPG test results. Finally, 3922 people were enrolled, including 2433 (62.0%) urban residents and 2195 (56.0%) women. The mean age was 58.47 ± 10.33 years, and 61.4% of them had senior high school and above education levels. A total of 941 (23.99%) had hypertension, 955 (24.35%) had dyslipidemia, 322 (8.21%) had a transient ischemic attack or stroke history, 29 (0.74%) had atrial fibrillation or valvular heart disease, and 81 (2.07%) had coronary heart disease. The general characteristics of participants are shown in Table 1.

**Table 1:** Baseline characteristics of the population.

Items	N (%)	Diabetes prevalence	Awareness	Treatment	Glycaemic control
Total	3922 (100.0)	609 (15.5)	501 (82.3)	223 (44.5)	104 (46.6)
**Residential areas**					
Urban	2433 (62.0)	346 (14.2)	321 (92.8)	80 (24.9)	39 (48.8)
Rural	1489 (38.0)	263 (17.7)	180 (68.4)	143 (79.4)	65 (45.5)
χ^2^		8.342	60.645	138.808	0.224
*p*		0.004	< 0.001	< 0.001	0.636
**Gender**					
Men	1727 (44.0)	255 (14.8)	218 (85.5)	80 (36.7)	42 (52.5)
Women	2195 (56.0)	354 (16.1)	283 (79.9)	143 (50.5)	62 (43.4)
χ^2^		1.367	3.126	9.54	1.723
*p*		0.242	0.077	0.002	0.189
**Age groups (years)**					
40–49	915 (23.3)	49 (5.4)	29 (59.2)	16 (55.2)	8 (50.0)
50–59	1376 (35.1)	212 (15.4)	179 (84.4)	45 (25.1)	21 (46.7)
60–69	993 (25.3)	196 (19.7)	161 (82.1)	90 (55.9)	42 (46.7)
70–79	496 (12.6)	126 (25.4)	110 (87.3)	62 (56.4)	30 (48.4)
≥ 80y	142 (3.6)	26 (18.3)	22 (84.6)	10 (45.5)	3 (30.0)
χ^2^		123.339	20.868	43.251	1.261
*P*		< 0.001	< 0.001	< 0.001	0.868
**Education level**					
Primary school and below	421 (10.7)	87 (20.7)	62 (71.3)	46 (74.2)	17 (37.0)
Junior school	1132 (28.9)	202 (17.8)	145 (71.8)	108 (74.5)	54 (50.0)
Senior school	1174 (29.9)	124 (10.6)	112 (90.3)	30 (26.8)	17 (56.7)
College and above	1195 (30.5)	196 (16.4)	182 (92.9)	39 (21.4)	16 (41.0)
χ^2^		35.868	43.023	128.362	3.929
*p*		< 0.001	< 0.001	< 0.001	0.269
**Annual income** (**RMB)**					
< 5000	515 (13.1)	101 (19.6)	72(71.3)	54 (75.0)	22 (40.7)
5000–10,000	439 (11.2)	76 (17.3)	51 (67.1)	43 (84.3)	25 (58.1)
10,000–19,999	418 (10.7)	68 (16.3)	55 (80.9)	39 (70.9)	18 (46.2)
≥ 20,000	2550 (65.0)	364 (14.3)	323 (88.7)	87 (26.9)	39 (44.8)
χ^2^		10.842	30.853	115.728	3.159
*p*		0.013	< 0.001	< 0.001	0.368
**Blood pressure level group**					
Normal	2981 (76.0)	415 (13.9)	353 (85.1)	107 (30.3)	51 (47.7)
Stage I	513 (13.1)	93 (18.1)	70 (75.3)	53 (75.7)	24 (45.3)
Stage II	285 (7.3)	61 (21.4)	47 (77.0)	37 (78.7)	17 (45.9)
Stage III	143 (3.7)	40 (28.0)	31 (77.5)	26 (83.9)	12 (46.2)
χ^2^		32.894	7.103	98.129	0.094
*p*		< 0.001	0.069	< 0.001	0.993
**Dyslipidaemia**					
Yes	955 (24.3)	264 (27.6)	216 (81.8)	127 (58.8)	60 (47.2)
No	2967 (75.7)	345 (11.6)	285 (82.6)	96 (33.7)	44 (45.8)
χ^2^		141.287	0.064	31.373	0.044
*p*		< 0.001	0.800	< 0.001	0.834
**BMI**					
Normal	1992 (50.8)	280 (14.1)	235 (83.9)	91 (38.7)	47 (51.6)
Overweight	1616 (41.2)	268 (16.6)	217 (81.0)	102 (47.0)	46 (45.1)
Obesity	314 (8.0)	61 (19.4)	49 (80.3)	30 (61.2)	11 (36.7)
χ^2^		8.303	0.996	9.275	2.214
*p*		0.016	0.608	0.01	0.331
**Smoking**					
Yes	452 (11.5)	81 (17.9)	64 (79.0)	42 (65.6)	21 (50.0)
No	3470 (88.5)	528 (15.2)	437 (82.8)	181 (41.4)	83 (45.9)
χ^2^		2.23	0.678	13.244	0.235
*P*		0.135	0.410	< 0.001	0.628
**Alcohol drinking**					
Yes	309 (7.9)	52 (16.8)	34 (65.4)	22 (64.7)	10 (45.5)
No	3613 (92.1)	557 (15.4)	467 (83.8)	201 (43.0)	94 (46.8)
χ^2^		0.433	11.106	6.023	0.014
*p*		0.511	0.001	0.014	0.907
**Physical exercise**					
Regular	2905 (74.1)	357 (12.3)	284 (79.6)	155 (54.6)	73 (47.1)
Inactivity	1017 (25.9)	252 (24.8)	217 (86.1)	68 (31.3)	31 (45.6)
χ^2^		89.585	4.357	26.902	0.043
*p*		< 0.001	0.037	< 0.001	0.835
**Salt intake**					
Less	132 (3.4)	32 (24.2)	28 (87.5)	23 (82.1)	13 (56.5)
Moderate	2270 (57.9)	205 (9.0)	144 (70.2)	87 (60.4)	41 (47.1)
More	1520 (38.8)	372 (24.5)	329 (88.4)	113 (34.3)	50 (44.2)
χ^2^		173.433	30.632	44.567	1.171
*p*		< 0.001	< 0.001	< 0.001	0.557
**Lean meat intake**					
Less	170 (4.3)	32 (18.8)	27 (84.4)	22 (81.5)	16 (72.7)
Moderate	3548 (90.5)	536 (15.1)	448 (83.6)	183 (40.8)	79 (43.2)
More	204 (5.2)	41 (20.1)	26 (63.4)	18 (69.2)	9 (50.0)
χ^2^		5.135	10.721	23.808	6.983
*p*		0.077	0.005	< 0.001	0.030
**Vegetable intake**					
< 5d/w	928 (23.7)	112 (12.1)	83 (74.1)	68 (81.9)	35 (51.5)
≥ 5d/w	2994 (76.3)	497 (16.6)	418 (84.1)	155 (37.1)	69 (44.5)
χ^2^		11.088	6.262	56.389	0.919
*p*		0.001	0.012	< 0.001	0.338
**Fruit intake**					
≤ 2d/w	380 (9.7)	78 (20.5)	66 (84.6)	49 (74.2)	27 (55.1)
3–4d/w	1138 (29.0)	139 (12.2)	104 (74.8)	81 (77.9)	36 (44.4)
≥ 5d/w	2404 (61.3)	392 (16.3)	331 (84.4)	93 (28.1)	41 (44.1)
χ^2^		17.874	6.846	106.628	1.811
*p*		< 0.001	0.033	< 0.001	0.404

Awareness rate: the proportion of people who were aware of diabetes among those with diabetes. Treatment rate: the proportion of people who received treatment among those who were aware of diabetes. Blood glucose control rate: the proportion of people whose blood glucose control reached the target level among those who received treatment.

*BMI* body mass index.

### Diabetes prevalence, awareness, treatment, FPG control rates

Among the 3922 respondents, there were 609 (15.5%) cases of diabetes. The diabetic prevalence rate in rural areas was higher than that in urban areas (17.7% vs*.* 14.2%, *p* = 0.004), while it did not significantly differ between men and women (14.8% in men vs*.* 16.1% in women, *p* = 0.242). Differences in the diabetic prevalence rate, associated with the participants’ age, educational levels, annual income, blood pressure, blood lipid levels, body mass index (BMI), exercise and dietary habits (taste, vegetables and fruit intake) (*p* < 0.05), were found. Among the respondents aged 40–79 years, the diabetic prevalence rate gradually rose with age [5.4% (40–49 years old) vs*.* 15.4% (50–59 years old) vs*.* 19.7% (60–69 years old) vs*.* 25.4% (70–79 years old), *p* < 0.001], blood pressure grade [13.9% (normal blood pressure) vs*.* 18.1% (grade I hypertension) vs*.* 21.4% (grade II hypertension) vs*.* 28.0% (grade III hypertension), *p* < 0.001] and BMI [14.1% (normal) vs*.* 16.6% (overweight) vs*.* 19.4% (obesity), *p* = 0.016]. The respondents with dyslipidemia had a significantly higher diabetic prevalence rate than those with a normal blood lipid level [27.6% (dyslipidemia) vs*.* 11.6% (normal blood lipid level), *p* < 0.001]. The diabetic prevalence rate was significantly higher in respondents who lacked exercise and preferred light or heavy taste than that in those who regularly exercised [24.8% (lack of exercise) vs*.* 12.3% (regular exercise), *p* < 0.001] and had moderate taste [24.2% (light taste) vs*.* 24.5% (heavy taste) vs*.* 9.0% (moderate taste), *p* < 0.001]. Besides, the diabetic prevalence rate negatively correlated with the increase in annual income [19.6% (< 5000 yuan) vs*.* 17.3% (5000–10,000 yuan) vs*.* 16.3% (10,000–19,999 yuan) vs*.* 14.3% (≥ 20,000 yuan), *p* = 0.013] (Table 1).

Among the 609 diabetic respondents, 501 (82.3%) cases were aware of diabetes. The diabetic awareness rate was higher in urban residents compared to that in rural residents (92.8% vs. 68.4%, *p* < 0.001), while it did not differ between men and women (85.5% in men vs*.* 79.9% in women, *p* = 0.077). Differences in the diabetic awareness rate, associates with the participants’ age, educational levels, annual income, drinking status, exercise, and dietary habits (taste, lean meat intake, vegetables, and fruit intake) (*p* < 0.05) (Table 1).

Among the 501 respondents who were aware of diabetes, 223 (44.5%) cases were treated with medication. The treatment rate was different between that in rural and urban areas, and between men and women (*p* < 0.05). The diabetic treatment rate was also different depending on age, educational level, annual income, blood pressure grade, blood lipid level, BMI and living habits (smoking, drinking, exercise and dietary habits) (*p* < 0.05) (Table 1).

Among the 223 diabetic respondents who were treated with medication, FPG was controlled in 101 (46.6%) cases (FPG < 7.0 mmol/L). The FPG control level was different between the respondents and depended on the different levels of meat intake (*p* = 0.03) (Table 1).

### Gender differences in diabetic prevalence, awareness, treatment, and control rates in different age groups

**Figure 1:**
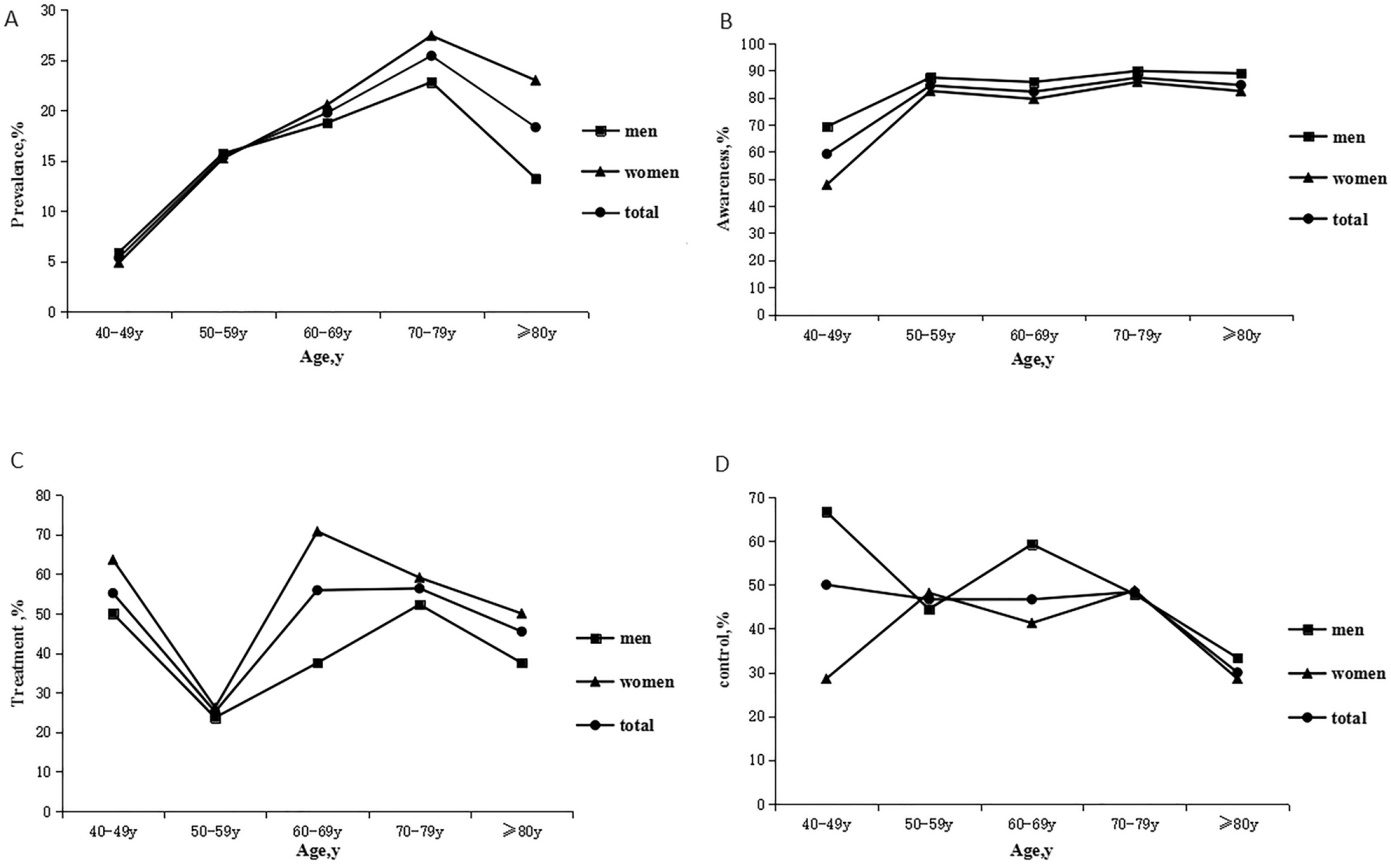
Gender differences in diabetic prevalence, awareness, treatment, and control rates in different age groups.

As shown in Fig. 1, the diabetic prevalence rate in men and women rose with age among the respondents aged 40–79 years. The diabetic prevalence rate in respondents under the age of 60 years was slightly higher in men than that in women, while it was significantly higher in women over the age of 60 years. The diabetic awareness rate of respondents over the age of 40 years was significantly higher in men than that in women, and the diabetic treatment rate of respondents over the age of 40 years was higher in women than that in men. Moreover, the diabetic control rate was slightly higher in women than that in men among the respondents aged 50–59 and 70–79 years, while it was obviously higher in men than that in women among the respondents aged 40–49, 60–69 and ≥ 80 years.

### Logistic regression analysis of influencing factors for diabetic prevalence, awareness, treatment, and control rates

#### Influencing factors for the diabetic prevalence rate

After correction of other factors, we found that age, hypertension, dyslipidemia, living habits (exercise habits) and dietary habits (taste), are related to the diabetic prevalence rate. The risk of developing diabetes among the respondents aged 50–59, 60–69, 70–79 and ≥ 80 years was respectively 2.55 times [odds ratio (OR) = 2.55, 95% confidence interval (95% CI) 1.82–3.56, *p* < 0.001], 2.90 times (OR = 2.90, 95% CI 2.06–4.10, *p* < 0.001), 3.31 times (OR = 3.31, 95% CI 2.28–4.82, *p* < 0.001) and 2.41 times (OR = 2.41, 95% CI 1.40–4.17, *p* = 0.002) that among those aged 40–49 years. The respondents with a higher hypertension grade also had an increased risk of developing diabetes (p = 0.022). The risk of developing diabetes among the respondents with dyslipidemia was 2.13 times that among those with a normal blood lipid level (OR = 2.13, 95% CI 1.75–2.60, *p* < 0.001). Regular exercise and moderate salt intake were protective factors against diabetes (*p* < 0.05) (Table 2).

**Table 2:** Multiple logistic regression analysis of influencing factors for diabetic prevalence, awareness, treatment, and control rates.

Items	Prevalence (n = 3922)		Awareness (n = 609)		Treatment (n = 609)		Glycaemic control (n = 609)	
	OR, 95% CI	*p*	OR, 95% CI	*p*	OR, 95% CI	***p***	OR, 95% CI	*p*
**Residential areas**								
Urban	1.00		1.00		1.00		1.00	
Rural	0.73 (0.46, 1.15)	0.173	0.09 (0.03, 0.27)	< 0.001	0.84 (0.34, 2.08)	0.708	0.54 (0.18, 1.61)	0.270
**Age groups (years)**								
40–49	1.00		1.00		1.00		1.00	
50–59	2.54 (1.82, 3.56)	< 0.001	3.64 (1.61, 8.25)	0.002	0.91 (0.42, 2.00)	0.818	0.90 (0.34, 2.38)	0.832
60–69	2.90 (2.06, 4.10)	< 0.001	4.14 (1.84, 9.31)	0.001	2.26 (1.06, 4.81)	0.035	1.68 (0.67, 4.21)	0.272
70–79	3.31 (2.28, 4.82)	< 0.001	5.34 (2.09, 13.69)	< 0.001	2.79 (1.24, 6.28)	0.013	2.00 (0.75, 5.35)	0.166
≥ 80	2.41 (1.40, 4.17)	0.002	2.40 (0.61, 9.46)	0.213	2.48 (0.81, 7.66)	0.113	1.16 (0.25, 5.33)	0.846
**Education level**								
Primary school and below	1.00		1.00		1.00		1.00	
Junior school	1.08 (0.77, 1.50)	0.675	1.65 (0.77, 3.52)	0.198	1.56 (0.78, 3.09)	0.208	2.11 (0.92, 4.83)	0.077
Senior school	0.50 (0.31, 0.81)	0.005	2.03 (0.60, 6.79)	0.253	1.00 (0.38, 2.63)	0.999	1.71 (0.52, 5.57)	0.377
College and above	0.84 (0.52, 1.36)	0.474	2.39 (0.69, 8.31)	0.172	0.80 (0.30, 2.10)	0.650	1.01 (0.30, 3.39)	0.991
**Annual income (RMB)**								
< 5000	1.00		1.00		1.00		1.00	
5000–10,000	1.08 (0.75, 1.54)	0.676	0.84 (0.39, 1.84)	0.670	1.36 (0.67, 2.79)	0.397	1.77 (0.80, 3.92)	0.159
10,000–19,999	1.24 (0.83, 1.85)	0.290	1.04 (0.41, 2.62)	0.934	1.50 (0.69, 3.28)	0.310	1.05 (0.44, 2.51)	0.922
≥ 20,000	1.33 (0.91, 1.96)	0.146	0.97 (0.41, 2.30)	0.945	0.97 (0.45, 2.06)	0.933	0.74 (0.29, 1.89)	0.533
**Blood pressure level group**								
Normal	1.00		1.00		1.00		1.00	
Stage I	1.13 (0.82, 1.57)	0.455	3.08 (1.48, 6.40)	0.003	1.71 (0.90, 3.26)	0.101	1.35 (0.65, 2.85)	0.423
Stage II	1.43 (0.97, 2.10)	0.069	3.43 (1.51, 7.79)	0.003	2.02 (0.95, 4.29)	0.067	1.52 (0.65, 3.54)	0.338
Stage III	1.96 (1.24, 3.09)	0.004	3.07 (1.19, 7.91)	0.020	2.39 (1.01, 5.63)	0.047	1.91 (0.74, 4.92)	0.181
**Dyslipidaemia**								
No	1.00		–	–	1.00		1.00	
Yes	2.13 (1.75, 2.60)	< 0.001	–	–	1.90 (1.28, 2.83)	0.001	1.65 (1.03, 2.65)	0.036
**BMI**								
Normal	1.00		–	–	1.00		–	–
Overweight	1.15 (0.94, 1.40)	0.172	–	–	1.06 (0.70, 1.61)	0.785	–	–
Obesity	1.16 (0.83, 1.62)	0.384	–	–	1.31 (0.66, 2.61)	0.440	–	–
**Physical exercise**								
Regular	1.00		1.00		1.00		1.00	
Inactivity	0.50 (0.41, 0.61)	< 0.001	1.08 (0.62, 1.87)	0.797	1.17 (0.76, 1.80)	0.476	1.17 (0.69, 1.98)	0.563
**Salt intake**								
Less	1.00		1.00		1.00		1.00	
Moderate	0.35 (0.21, 0.56)	< 0.001	0.22 (0.06, 0.76)	0.017	0.54 (0.20, 1.43)	0.214	0.74 (0.29, 1.90)	0.535
Higher	0.81 (0.50, 1.31)	0.381	0.47 (0.13, 1.65)	0.239	0.54 (0.20, 1.41)	0.206	0.64 (0.25, 1.62)	0.345
**Vegetable intake**								
< 5d/w	1.00		1.00		1.00		1.00	
≥ 5d/w	1.18 (0.84, 1.67)	0.342	1.85 (0.79, 4.30)	0.155	1.25 (0.61, 2.53)	0.543	1.09 (0.50, 2.39)	0.831
**Fruit intake**								
≤ 2d/w	1.00		1.00		1.00		1.00	
3–4d/w	0.95 (0.66, 1.35)	0.795	0.58 (0.23, 1.43)	0.233	0.86 (0.43, 1.72)	0.667	0.55 (0.26, 1.16)	0.116
≥ 5d/w	0.99 (0.70, 1.40)	0.941	0.22 (0.09, 0.56)	0.001	0.31 (0.16, 0.60)	0.001	0.26 (0.12, 0.57)	0.001

#### Influencing factors for the diabetic awareness rate

After correction of other factors, we found that the region, age, hypertension, and dietary habits (fruit and salt intake), are related to the diabetic awareness rate. The higher age and blood pressure grade corresponded to a higher diabetic awareness rate (*p* < 0.05). The diabetic awareness rate was lower among the respondents who lived in rural areas, ate more fruit on a weekly basis, and moderately consumed salt (*p* < 0.05) (Table 2).

#### Influencing factors for the diabetic treatment rate

After correction of other factors, we found that age, dyslipidemia, and dietary habits (fruit intake), were related to the diabetic treatment rate. Among the diabetic respondents who were older and had dyslipidemia, the diabetes treatment rare was higher (*p* < 0.05). The rate was lower among the respondents who ate more fruit on a weekly basis (*p* < 0.05) (Table 2).

#### Influencing factors for the diabetic control rate

After correction of other factors, we found that dyslipidemia and dietary habits (fruit intake), were related to the FPG control rate. This rate was higher among the respondents with dyslipidemia than that among those with a normal blood lipid level (OR = 1.65, 95% CI 1.03–2.65, *p* = 0.036). Weekly consumption of fruit led to a lower FPG control rate (*p* = 0.003) (Table 2).

### Differences in gender, hypertension, dyslipidemia, and BMI among the diabetic respondents in urban and rural areas

The proportion of hypertension [81.0% (rural areas) vs*.* 41.0% (urban areas), *p* < 0.001], dyslipidemia [47.9% (rural areas) vs*.* 39.9% (urban areas), *p* = 0.048], and overweight or obesity [normal weight: 34.2% (rural areas) vs*.* 54.9% (urban areas), *p* < 0.001] among rural areas’ diabetic respondents were far higher than those in urban areas (Table 3).

**Table 3:** Differences in gender, hypertension, dyslipidemia. and BMI among the diabetic respondents in urban and rural areas.

Items	Urban	Rural	Total	χ^2^	*p*
**Gender**	346 (100.0)	263 (100.0)	609 (100.0)	13.458	< 0.001
Men	167 (48.3)	88 (33.5)	255 (41.9)		
Women	179 (51.7)	175 (66.5)	354 (58.1)		
**Hypertension**				98.080	< 0.001
Yes	142 (41.0)	213 (81.0)	355 (58.3)		
No	204 (59.0)	50 (19.0)	254 (41.7)		
**Dyslipidaemia**				3.918	0.048
Yes	138 (39.9)	126 (47.9)	264 (43.3)		
No	208 (60.1)	137 (52.1)	345 (56.7)		
**BMI**				35.574	< 0.001
Normal	190 (54.9)	90 (34.2)	280 (46.0)		
Overweight	138 (39.9)	130 (49.4)	268 (44.0)		
Obesity	18 (5.2)	43 (16.3)	61 (10.0)		

## Discussion

In this study, the diabetic prevalence, awareness, treatment, control rates, and their influencing factors, were explored in 2019 and for the first time, among adults aged 40 years and above in Shenyang, China. A total of 3922 participants were enrolled, and the diabetic overall prevalence rate was 15.5%. According to a national survey in China in 2010, the diabetic prevalence, awareness, treatment and control rates in Chinese adults, were 11.6%, 30.1%, 25.8% and 39.7%, respectively^7^. In this study, the diabetic prevalence rate (15.5%) was higher than that of the 2010 national level. Although its awareness (82.3%) and treatment (36.6%) rates were higher than those of the national level, its control rate (17.1%) was markedly lower than that of the national level. It can be seen that the diabetic control task in Shenyang, China, remains formidable, and more attention should be paid to raising the diabetic control rate and lowering its prevalence rate, while further increasing the diabetic awareness and treatment rates. Chinese people's living standards have been continuously improving and the average life expectancy has been constantly increasing, which may be related to the increase in the diabetic overall prevalence rate^8,9^. In addition, the respondents in this survey were people aged 40 years and above, living in Shenyang, whereas general people aged above 18 years, living in communities or townships, were mostly taken as subjects of a previous study^10,11^. Therefore, the presence of different respondents may also be one of the reasons for the high diabetic prevalence rate in this survey.

According to the survey results, the diabetic prevalence rate was higher in rural areas compared to that in urban areas (17.7% vs*.* 14.2%, *p* = 0.004), while the diabetic awareness rate was lower in rural areas compared to that in urban areas (68.4% vs*.* 92.8%, *p* < 0.001). The above findings suggest that it is necessary to strengthen adults’ diabetic screening in low economic level rural areas.

In this study, the diabetic prevalence rate was 82.3%, which is far higher than that in other Chinese regions and foreign countries (Shanghai: 28.06%^12^, Shandong: 34.8%^13^, Jiangsu: 58.35%^14^, Jilin: 64.1%^15^, Zhejiang: 59.19%^16^, Rural Diab study: 60.11%^17^, Switzerland: 65.3%^18^). This rate should relate to the differences in the study year, and health education status in different regions and countries. In addition, this may also be due to the relatively lower proportion of rural population (1/3) in this study. The diabetic awareness rate among urban residents was higher than that among rural residents (92.8% vs*.* 68.4%, *p* < 0.001), which may be due to the limited health knowledge that is received in rural areas^19,20^. There is a study revealed that the increases in age and risks of developing hypertension and dyslipidemia, will correspondingly increase the opportunity of receiving disease-related medical education, which will improve diabetic awareness and treatment rates^10^.

In the present study, the diabetic treatment rate in rural areas, was higher than that in urban areas (79.4% vs*.* 24.9%, *p* < 0.001). There are studies showing that the better the individual's self-reported health conditions are, the less likely the diabetic medication would be is administered, and that such influence is greater in urban areas^21^. In a clinical study that was performed by this research group, it was found that some urban residents were more willing to control blood glucose through exercise and diet control, that may explain the lower diabetic treatment rate in urban areas, and which should arouse the attention of researchers. Currently, there have been no relevant literature reports. In this study, the proportion of metabolic syndromes, such as hypertension, dyslipidemia, overweight or obesity among the diabetic respondents in rural areas was markedly higher than that among those in urban areas[hypertension: 81.0% (rural areas) vs*.* 41.0% (urban areas), *p* < 0.001; dyslipidemia: 47.9% (rural areas) vs*.* 39.9%(urban areas), *p* = 0.048; overweight or obesity: 65.7% (rural areas) vs*.* 45.1% (urban areas), *p* < 0.001] (Table 3). This may be another important reason for the higher diabetic treatment rate areas compared to that in urban areas^10^.

This study also revealed that the diabetic control rate in Shenyang was 17.1% higher than that in Shanghai (12.42%)^12^, Shandong (11.5%)^13^ and Jiangsu (14.12%)^14^, but lower than that in Jilin (23.3%)^15^, Zhejiang (23.87%)^16^ and Rural Diab study (18.77%)^17^. The potential reasons may be associated with differences in the definition or diagnostic criteria of diabetes dietary habits^7,10,17^, economic and educational levels, and degrees of emphasis on the publicity on diabetic prevention and control among the regions^22^.

There are few studies on gender differences in diabetic prevalence, awareness, treatment and control rates. In this study, the diabetic awareness rate was significantly higher in men than that in women (Fig. 1). A study has demonstrated that the women diabetic awareness rate is higher than in men in rural areas^21^, which is different from the results reported in this study. This difference may be due to the larger urban population that was included in this study. Besides, the diabetic control rate was also different due to differences in gender and age groups. According to the survey results, the diabetic control rate was slightly higher in women than that in men at the age of 50–59 and 70–79 years (Fig. 1), while it was obviously higher in men at the age of 40–49, 60–69 and ≥ 80 years. It was previously found that women aged 55–64 years have a lower diabetic control rate than that of same age men in rural areas (13.1% vs*.* 31.0%, *p* = 0.042)^23^, suggesting the necessity of enhancing women diabetic education, awareness and control rates in the future.

Advanced age, a low educational level, lack of exercise, hypertension, and dyslipidemia, are risk factors for diabetes^9,24^. In this study, it was also confirmed that advanced age, hypertension and dyslipidemia were influencing factors for diabetes, indicating that the comprehensive management strategy for diabetes should cover the control of body weight, blood pressure and blood lipids. Research has argued that raising the educational level, moderate physical activity and good control of blood pressure and blood lipids can lower the risk of diabetes^17^.

The relation between drinking and diabetes remains controversial. According to a meta-analysis, light to moderate alcohol consumption leads to a lower risk of diabetes, while heavy alcohol consumption raises the risk of diabetes in men^17^. In this study, a relationship between alcohol consumption and diabetes was not found, which may be due to the lack of subdivided into different grades. Previous studies have shown that smoking is a risk factor for diabetes, and that the larger the cumulative smoking amount, the higher the risk of diabetes^25^. This study did not prove that smoking was related to diabetes, which may be due to the misclassification of former smokers as non-smokers.

Several studies have indicated that an unhealthy diet raises the risk of diabetes^9,10^, and that patients' dietary habits are altered after developing diabetes^26^. Kim et al.^26^ argued that pre-diabetic and diabetic patients tend to eat less sugar, fat, and carbohydrates, and eat more fruit. In this study, the results showed that the diabetic respondents, who consumed fruit for ≥ 5 days/week, account for 16.3%, had lower diabetic treatment (28.1%) and control (44.1%) rates, but the types of fruit were not further investigated. A study has shown that healthy people who keep the habit of consuming fresh fruit, have a much lower diabetic risk, and that the death rate of patients who consume fruits for ≥ 3 days/week, declines by 17% compared with that of diabetic patients who consume very little fruit^27^.

There were imitations in this study. First, the study population was recruited from Shenyang, China, and they were aged ≥ 40 years, therefore, the research results failed to represent the epidemiology of diabetes in the Chinese population. Second, the cross-sectional design may have led to selection bias. Third, only FPG was used as a diabetes index for all respondents, the glucose tolerance test lacked, and the level of glycated hemoglobin was not detected, thus, the diabetic prevalence rate may be underestimated.

In this study, the diabetic prevalence, awareness, treatment, control rates and their related risk factors were reported in Shenyang, China in 2019. It was confirmed that the diabetic prevalence was higher, while the treatment and control rates were lower in Shenyang. Therefore, it is necessary to reduce the impact of related risk factors to lower the diabetic burden.

## Research methods

### Study participants

Based on the National Health Commission’s public welfare project "Screening and Intervention of High-Risk Stroke Population in 2018", this survey was conducted on permanent residents (lived locally for 6 months or more) aged ≥ 40 years at the screening points of communities and townships through multi-stage cluster random sampling from April to May 2019. In the first stage, 2 survey sites were selected from the geographic area: Fengle Subdistrict, Dongling District, northeastern Shenyang (a middle-economic-level area), and Linshengbao Town, Sujiatun District, southwestern Shenyang (a middle-low-economic-level area). In the second stage, 8 communities and 8 village committees were randomly selected from the Fengle Subdistrict and Linshengbao Town, respectively. In the third stage, the respondents were selected from all adults aged ≥ 40 years in each community/village committee through simple random sampling. Pregnant women and people with mental disorders were excluded. If someone refused to participate in the research, or the data could not be collected for various reasons, other adults aged ≥ 40 years, in the nearest community or village, would have been selected as substitutes, thus ensuring sufficient samples.

**Figure 2:**
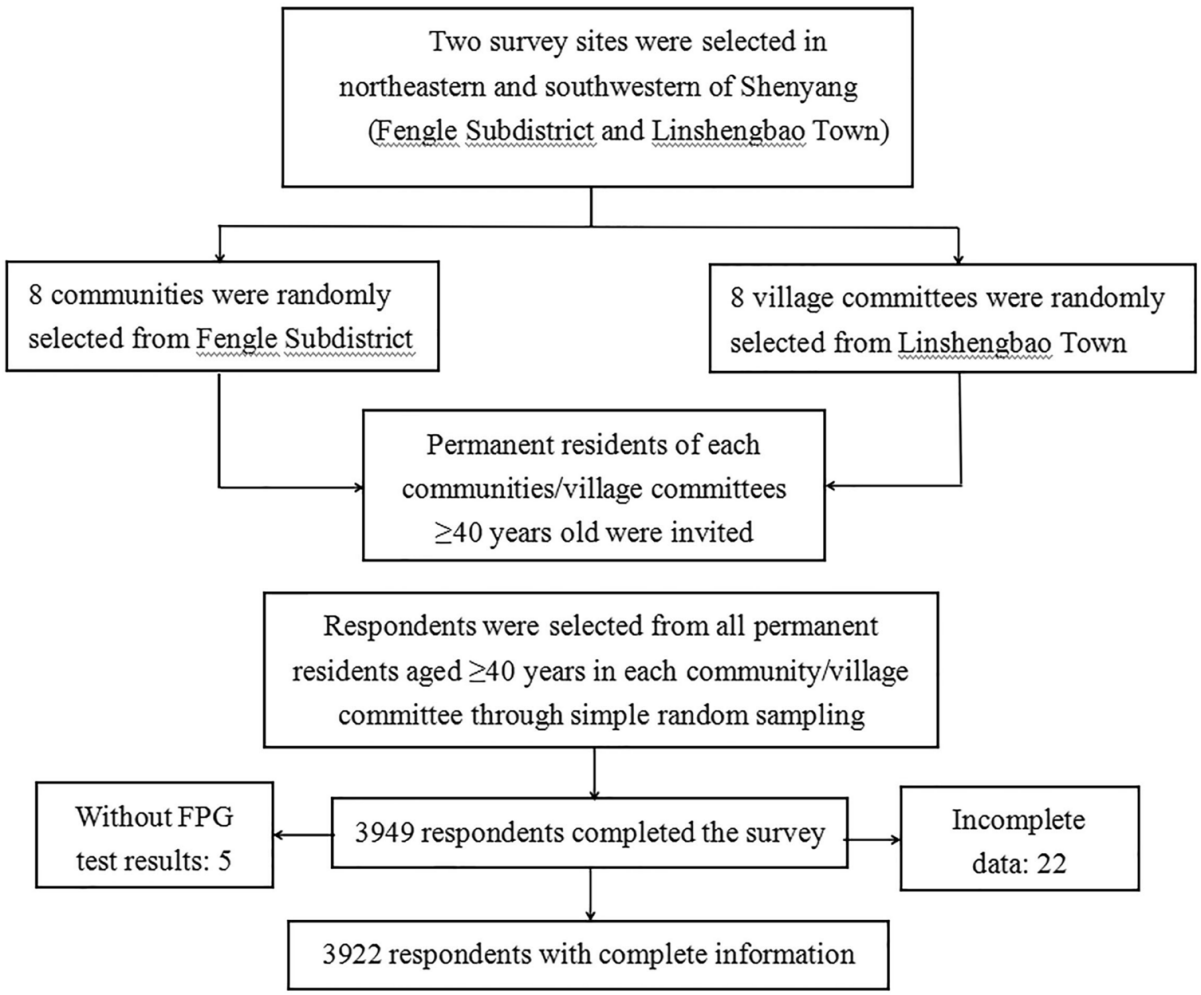
The study sampling process.

In this study, it was planned to enroll 4000 adults aged ≥ 40 years old in Shenyang, Liaoning, China, including 2400 in urban areas (Fengle Subdistrict) and 1600 participants in rural areas (Linshengbao Town). However, a total of 3949 people agreed to participate, including 22 with incomplete data and 5 without FPG test results. Finally, a total of 3922 people were enrolled, including 2433 (62.0%) urban residents and 2195 (56.0%) women (Fig. 2).

Upon approval by the Stroke Prevention Project, National Health Commission, this study was conducted according to the protocol of "Screening and Intervention of High-Risk Stroke Population in 2018". The health-related information, physical examination and laboratory examination collected during the screening of this project did not pose any risk of injury to the investigated subjects. Venous blood sampling may cause temporary mild pain and subcutaneous stasis, which has been orally informed. The relevant information exposed in the study was implemented in accordance with the ethical norms proposed by the Brain prevention Commission of the National Health and Family Planning Commission (see "Technical plan of 2018 stroke High-risk Population Screening and Intervention Project 4. Ethical issue"). Before the survey, the statutory guardians signed the informed consent on behalf of the illiterate participants, and all other participants personally signed the informed consent. The number of people, who were screened in all communities and townships, reached more than 85% of the screening subjects at each screening point, and the used household registration information for the sampling came from the government departments.

### Collection of information

Face-to-face questionnaire surveys, physical and laboratory examinations were performed by well-trained investigators using the standard technical solution. The survey questionnaire covered basic demographic information, such as the name, gender, date of birth, educational level, annual income and living habits. The respondents were divided into 5 age groups: 40–49 years old, 50–59 years old, 60–69 years old, 70–79 years old and ≥ 80 years old. For the educational level, the respondents were divided into 4 groups, according to the years of formal education: illiteracy^28^/primary school, junior high school, senior high school, bachelor’s degree and above. The personal medical history was obtained through the patient's self-report or medical records, including hypertension, diabetes, and dyslipidemia. Lifestyle characteristics included exercise habits, smoking, drinking, and dietary habits (taste, lean meat intake, vegetable intake, fruit intake).

### Physical examination and definition of related diseases

Physical examination items included blood pressure (BP, including systolic and diastolic blood pressures) at rest (rest for at least 20 min), height and weight without shoes. The body mass index (BMI) was calculated as follows: BMI = body weight (kg)/height (m^2^). Besides, after fasting for at least 8 h, venous blood was collected by the medical workers of The People's Hospital of Liaoning Provincial, and then the FPG was detected using a biochemical analyzer.

Hypertension^29^ could be diagnosed if any one of the following 2 conditions was met: (1) If taking no antihypertensive drugs, the patient had the mean systolic blood pressure of ≥ 140 mmHg and/or the mean diastolic blood pressure was ≥ 90 mmHg in two measurements on the spot at rest (rest for at least 20 min); and (2) if the hypertension diagnosed by the doctor was reported by the patient, and/or the patient was taking antihypertensive drugs in the past 2 weeks. Dyslipidemia was defined as follows: a history of dyslipidemia was reported by the patient or the patient was taking lipid-lowering drugs for at least 2 weeks currently. BMI < 24 kg/m^2^ is normal, 24 kg/m^2^ ≤ BMI < 28 kg/m^2^ indicates overweight, and BMI ≥ 28 kg/m^2^ indicates obesity^30^. Exercise habits were defined as follows: a regular exercise corresponded to a moderate-intensity exercise that is equivalent to fast-walking, ≥ 3 times per week and for ≥ 30 min, including moderate and heavy manual labor; and a lack of exercise corresponded to the absence of exercise as defined the above criteria. Smoking was defined as > 1 cigarette/day for at least half a year^31^. Drinking was defined as a minimum of one glass of wine, consumed in the last 30 days. Vegetable intake corresponded to 300 g of vegetables/day and fruit intake, to 200 g of fruit/day.

Diabetes^32^ could be diagnosed if any one of the following two conditions was met: (1) venous blood FPG of ≥ 7.0 mmol/L; and (2) the patient had a clear diabetic history, and/or, was taking hypoglycemic drugs. Diabetic awareness was defined as diabetes that was diagnosed by the doctor and previously reported by the patient. Diabetic treatment was defined as patients with known diabetes, who took at least one prescription drug and for at least 2 weeks. The diabetic control corresponded to an FPG of the treated diabetic patients that was < 7.0 mmol/L.

### Quality control and statistical methods

The accuracy of all case information was reviewed and controlled by the quality control group members, and a diagnosis was made by the group experts. The questionnaire data were sorted, proofread, and entered using the EpiData3.1 software. SPSS25.0 was used for analysis, and *p* < 0.05 of two-sided test was considered statistically significant. Continuous variables were expressed as mean ± standard deviation (χ ± s), and *t* test was performed for the comparison between two groups. Besides, categorical variables were expressed as rate (%), and chi-square test (chi-square test for four-fold table data and R × C table data) was performed for the comparison between two groups and among three groups. Multivariate logistic regression analysis was adopted for the diabetic risk factors (*p* < 0.1 in univariate analysis), and the results were expressed as odds ratio (OR) with 95% confidence interval (CI).
